# The novel IGF-IR/Akt–dependent anticancer activities of glucosamine

**DOI:** 10.1186/1471-2407-14-31

**Published:** 2014-01-20

**Authors:** Ki-Hoon Song, Ju-Hee Kang, Jong-Kyu Woo, Jeong-Seok Nam, Hye-Young Min, Ho-Young Lee, Soo-Youl Kim, Seung-Hyun Oh

**Affiliations:** 1 Research Institute, National Cancer Center, Goyang-si, Gyeonggi-do 410-769, Republic of Korea; 2 Department of Food and Nutrition, College of Human Ecology, Chung-Ang University, Ansung, Gyeonggi-do, Republic of Korea; 3 Laboratory of Tumor Suppressor, Lee Gil Ya Cancer and Diabetes Institute, Gachon University, Incheon 406-840, Republic of Korea; 4 College of Pharmacy, Seoul National University, Seoul 151-742, Republic of Korea; 5 Gachon Institute of Pharmaceutical Science, Gachon University, Incheon 406-840, Republic of Korea; 6 College of Pharmacy, Gachon University, 7-45 Songdo-dong, Yeonsu-gu, Incheon 406-840, Republic of Korea

**Keywords:** Glucosamine, Anticancer agent, IGF-1R, Akt, Glycosylation, ER-stress

## Abstract

**Background:**

Recent studies have shown that glucosamine inhibits the proliferation of various human cancer cell lines and downregulates the activity of COX-2, HIF-1α, p70S6K, and transglutaminase 2. Because the IGF-1R/Akt pathway is a common upstream regulator of p70S6K, HIF-1α, and COX-2, we hypothesized that glucosamine inhibits cancer cell proliferation through this pathway.

**Methods:**

We used various *in vitro* assays including flow cytometry assays, small interfering RNA (siRNA) transfection, western blot analysis, MTT (3-(4,5-dimethylthiazol-2-yl)-2,5-diphenyltetrazolium bromide) assays, reverse transcription-polymerase chain reaction, and *in vivo* xenograft mouse model to confirm anticancer activities of glucosamine and to investigate the molecular mechanism.

**Results:**

We found that glucosamine inhibited the growth of human non-small cell lung cancer (NSCLC) cells and negatively regulated the expression of IGF-1R and phosphorylation of Akt. Glucosamine decreased the stability of IGF-1R and induced its proteasomal degradation by increasing the levels of abnormal glycosylation on IGF-1R. Moreover, picropodophyllin, a selective inhibitor of IGF-1R, and the IGF-1R blocking antibody IMC-A12 induced significant cell growth inhibition in glucosamine-sensitive, but not glucosamine-resistant cell lines. Using *in vivo* xenograft model, we confirmed that glucosamine prohibits primary tumor growth through reducing IGF-1R signalling and increasing ER-stress.

**Conclusions:**

Taken together, our results suggest that targeting the IGF-1R/Akt pathway with glucosamine may be an effective therapeutic strategy for treating some type of cancer.

## Background

Since the effect of glucosamine as an inhibitor of tumor growth was first reported by Quastel and Cantero, [1] many *in vitro* studies have shown that it interferes with the glycoslyation of glycoproteins, [2,3] decreases the rate of glycolysis and fructolysis, [4,5] and changes the component ratio of nucleotides in various carcinoma cell lines [6,7]. Results of a recent study indicated that glucosamine induces G1 cell-cycle arrest in mesangial cells and human cancer cells through a mechanism involving decreased expression of cyclin D1 and increased expression of p21^Waf1/Cip1^, which are positive and negative regulators of cell cycle progression, respectively [8,9].

The PI3K/Akt pathway is often overactivated in various types of cancer cells. PI3K/Akt can transmit signals from RTKs and G-protein-coupled receptors that are activated by growth factors or cytokines; therefore, the PI3K/Akt signal transduction pathway regulates multiple cellular functions, including transcription, translation, and cell proliferation, cell cycle progression, and survival [10-12]. Although the RTK-mediated signal transduction pathways overlap, PI3K-mediated activation of Akt specifically contributes to the anti-apoptotic activity of IGF-1R.

Recent studies have demonstrated that target proteins of glucosamine may exist in cancer cells [13-16]. Glucosamine inhibits the growth of cancer cells by downregulating the phosphorylation of p70S6K, a regulator of protein translation [15]. In addition, glucosamine inhibits HIF-1α by inhibiting protein translation through the reduction of phosphorylated p70S6K levels [16]. Jang et al. reported that glucosamine hydrochloride inhibits N-glycosylation of COX-2 and enhances COX-2 protein turnover [13]. Finally, glucosamine induces NF-κB inactivation by inhibiting transglutaminase 2 (TGase 2) activity [14]. Together, these studies suggest that glucosamine has potential as an anticancer drug, although its mechanism of action remains poorly understood [17]. Thus, we tested whether the IGF-1R/PI3K/Akt pathway, upstream of p70S6K and COX-2, is target of glucosamine. We also investigated the molecular mechanisms underlying the anticancer activity of glucosamine in NSCLC cells.

## Methods

### Cell lines and materials

Human NSCLC cell lines A549, H226B, H1299, and H460 were purchased from the American Type Culture Collection (Manassas, VA, USA).

The HA-Akt1 (T308D/S473D) expression vector was kindly provided by Dr. Gordon Mills (The University of Texas MD Anderson Cancer Center). The H226B-Babe cells were produced by infecting H226B NSCLC cells with a pBabe retroviral control vector. The H226B-Akt1-DD cells that possess a constitutively active form of Akt were produced by infecting H226B with a pBabe-HA-Akt1-DD construct harboring mutations that change Ser473 and Thr308 to aspartic acids. The H226B-Akt2-DD and The H226B-Akt3-DD cells were kindly provided by Dr. Ho-Young Lee (College of Pharmacy, Seoul National University, Seoul, Republic of Korea).

D-(+)-Glucosamine hydrochloride, MG132, and tunicamycin (TN) were purchased from Sigma-Aldrich (St Louis, MO, USA). Antibodies against pIGF-1R, pAkt, pERK1/2, Akt, PTEN, PARP, PDI, IRE1α, ATF4, GRP78, CHOP, and a/β-tubulin were purchased from Cell Signaling Technology (Beverly, MA, USA). Antibodies against IGF-1R, COX-2, CDK2, CDK4, and β- ACTIN were purchased from Santa Cruz Biotechnology, Inc. (Santa Cruz, CA, USA), and the antibody against TGase 2 was obtained from Thermo Fisher Scientific, Inc. (Fremont, CA, USA).

### Xenograft mouse tumor model

All animal experimental procedures were approved by Institutional Animal Care and Use Committee (IACUC) of National Cancer Center in Republic of Korea. To confirm antitumor effect of glucosamine in animal, we used xenograft tumor model. A549 cells (5 x 10^6^ cells) were subcutaneously injected into flank region of BALB/c nude mice. After cancer cell injection, glucosamine (500 mg/kg body weight/day) was administered intrapenitorially to immuoncompromised mice. Tumor volume was measured using caliper and calculated according to the formula (*L x W*^*2*^)/2. All of the mice were sacrificed on Day 71, and tumor tissues were isolated from them. The results were represented as the mean of tumor volumes (n = 10) with SEM.

### siRNA transfection

For RNA interference, A549, H1299, and H460 cells transfected with 40 nM siRNA. Double-stranded siRNAs designed to target *IGF-1R* (5′-CUG ACA UGG GCC UUU AAG A-3′), and a scrambled non-targeting siRNA were synthesized by Bionner (Seoul, South Korea). Cells were transfected with siRNAs using Lipofectamine reagent (Invitrogen, CA, USA) according to the manufacturer’s protocol.

### Semiquantitative RT-PCR

First strand cDNA was synthesized from 2 μg of extracted RNA using M-MLV reverse transcriptase (Invitrogen). RT-PCR was carried out with gene-specific primers for *IGF-1R, COX-2*, *XBP1, GRP78, CHOP, ATF4, GAPDH,* and *β-ACTIN* (Table 1). Primers amplifying a region of *β-ACTIN* or *GAPDH* were used as an internal control.

**Table 1:** Primer sequences used for RT-PCR

**Gene**	**Primer sequence (5′ - 3′)**
*IGF-1R*	F 5′-ACG CCA ATA AGT TCG TCC AC-3′
	R 5′-TCC ATC CTT GAG GGA CTC AG-3′
*COX-2*	F 5′-ATC TTT GGG GAG ACC ATG GTA GA-3′
	R 5′-ACT GAA TTG AGG CAG TGT TGA TG-3′
*XBP1*	F 5′-TTA CGA GAG AAA ACT CAT GGC C-3′
	R 5′-GGG TCC AAG TTG TCC AGA ATG C-3′
*GRP78*	F 5′-GGT ACA TTT GAT CTG ACT G-3′
	R 5′-CAC TTC ACT AGA GTT TGC TG-3′
*CHOP*	F 5′-CTT CAC TAC TCT TGA CCC TGC AT-3′
	R 5′-ATG TGC ACT GGA GAT TGC TT-3′
*ATF4*	F 5′-GTT CTC CAG CGA CAA GGC TA-3′
	R 5′-ATC CTC CTT GCT GTT GTT GG-3′
*GAPDH*	F 5′-GGT GAA GGT CGG TGT GAA CGG ATT T-3′
	R 5′-ATT GCC AAA GTT GTC ATG GAT GAC C-3′
*β-ACTIN*	F 5′-GTG GGG CGC CCC AGG CAC CA-3′
	R 5′-CTC CTT AAT GTC ACG CAC GAT TTC-3′

### Western blot analysis

Preparation of whole-cell lysates from cancer cells, electrophoresis, and membrane transfer were performed as previously described [18]. The membranes were then incubated overnight at 4°C with primary antibodies in TBS-T containing 5% bovine serum albumin. Membranes were washed with TBS-T and then incubated with an appropriate horseradish peroxidase-conjugated secondary antibody in 5% skim milk for 1 hour at room temperature.

### Cell proliferation analysis

To determine the effects of glucosamine on the proliferation of various cancer cell lines, cells were seeded in 96-well plates (3,000 cells/well). On the following day, the medium was replaced with medium containing glucosamine, picropodophyllin, and A12 at the desired concentrations. After incubation for an additional 2 days, MTT assay was performed according to standard procedures. The bars represent SD of results.

### Cell cycle analysis

For the cell cycle analysis, three human NSCLC cell lines were treated with the indicated concentration of glucosamine. Floating and attached cells were fixed in 70% ethanol for 1 hour at 4°C. After centrifugation, the cell pellet was washed twice with phosphate-buffered saline (PBS) and stained with propidium iodide (PI) containing RNase A (40 μg/ml) for 30 minutes at 4°C in the dark. The total cellular DNA content of each cancer cell line was quantified by flow cytometry.

### Apoptosis analysis

To analyze the number of apoptotic cells after 2 days of glucosamine treatment, A549, H1299, and H460 cells were harvested and washed twice with PBS on ice. The cells were resuspended in 1 X binding buffer containing 5 μl fluorescein isothiocyanate (FITC)-conjugated Annexin V and 5 μl PI. Apoptotic events were detected by flow cytometry at 488 nm and 633 nm using the FITC Annexin V apoptosis detection kit I (BD Pharmingen, San Jose, CA). All procedures were carried out according to the manufacturer’s instructions.

### Immunohistochemistry

Primary tumors from PBS or glucosamine treated animals were embedded in paraffin depending on the application. The 5 μm tumor tissue sections were prepared for immunohistochemistry. Paraffin sections were incubated overnight at 4°C with primary antibody against anti-phospho-Akt (Cell Signaling Technology; 1 : 100 dilution) and then processed for avidin – biotin immunohistochemistry according to the manufacturer’s instructions (Vector Laboratories, Burlingame, CA). These sections counterstained with hematoxylin and eosin-Y (H&E). Immunohistochemical analysis were performed as previously described [18].

## Results

### Glucosamine induces cell cycle arrest and apoptosis in NSCLC cells

**Figure 1:**
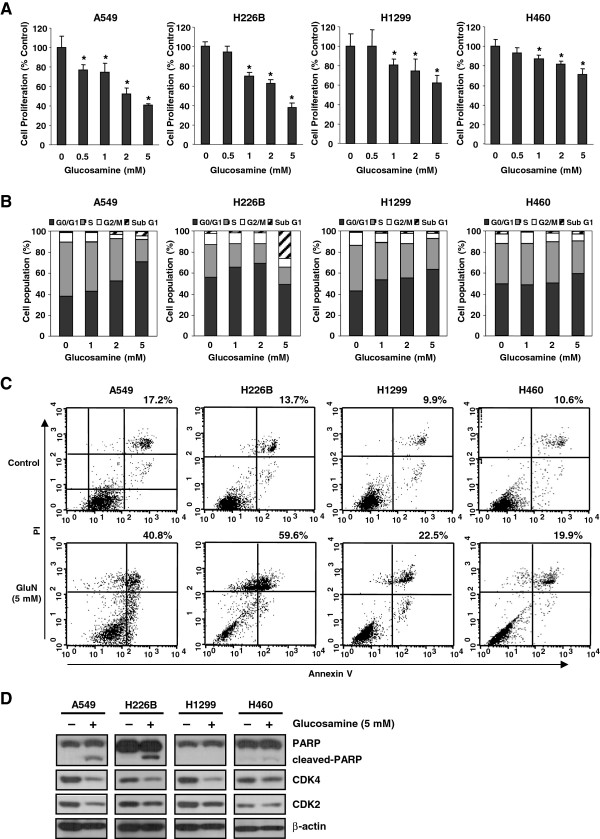
**Effect of glucosamine on cell growth, cell cycle arrest, and apoptosis in NSCLC cells. (A)** MTT growth assay of A549, H1299, and H460 cells. *P < 0.05 compared with glucosamine 0 mM. **(B)** NSCLC cells were treated with glucosamine for 2 days and then stained with PI for cell-cycle analysis. **(C)** Apoptosis/necrosis was determined by Annexin V-FITC and PI staining. **(D)** The protein levels of CDK2, CDK4, PARP, and cleaved PARP.

Previous studies have reported that glucosamine inhibits cell growth [15,16] and cell-cycle progression [8,9,19] and induces apoptosis [20] in various cell lines. We therefore investigated whether the anti-cancer effect of glucosamine was associated with cell growth, cell-cycle arrest and apoptosis in NSCLC cell lines. Glucosamine reduced the proliferation of all four NSCLC cell lines, but the extent of the inhibition differed among NSCLC cell lines (Figure 1A). Flow cytometric analysis indicated that glucosamine induced cell-cycle arrest at the G0/G1 phase in a dose-dependent manner (Figure 1B) and that glucosamine induced apoptosis in A549, H226B, H1299, and H460 NSCLC cell lines (Figure 1C and Additional file 1: Figure S1). Consistent with the results of the cell proliferation assay, in the cell cycle and apoptosis analyses, the A549 and H226B cells had a more significant response to glucosamine than the others.

In addition, expression of cleaved poly-(ADP-ribose) polymerase (PARP), a marker for apoptosis, was high in A549 and H226B cells and low in H460 cells (Figure 1D). Treatment with 5 mM glucosamine reduced the expression of both CDK4 and CDK2 in A549 and H226B cells and that of CDK4 only in H1299 cells. In contrast, the levels of CDK4 and CDK2 were not obviously changed in H460 cells (Figure 1D). These findings suggest that the glucosamine-mediated growth inhibition of NSCLC cells is associated with the induction of cell-cycle arrest and apoptosis.

### The basal expression levels of TGase 2 and COX-2 proteins in NSCLC cells are not correlated with glucosamine sensitivity

We investigated the expression levels of TGase 2 and COX-2 proteins that were previously identified as major targets of glucosamine. Expression of TGase 2 was markedly higher in A549 and H1299 cells than in H460 and H226B cells. We also found that A549 and H460 cell lines showed a high basal level of COX-2 expression, whereas COX-2 expression was not detected in H1299 cells (Additional file 2: Figure S2). Therefore, the basal TGase 2 and COX-2 levels in the NSCLC cell lines were not correlated with glucosamine sensitivity.

### Glucosamine suppresses activation of Akt by reducing IGF-1R expression in cell lines that have an IGF-1R-dependent Akt activation pathway

**Figure 2:**
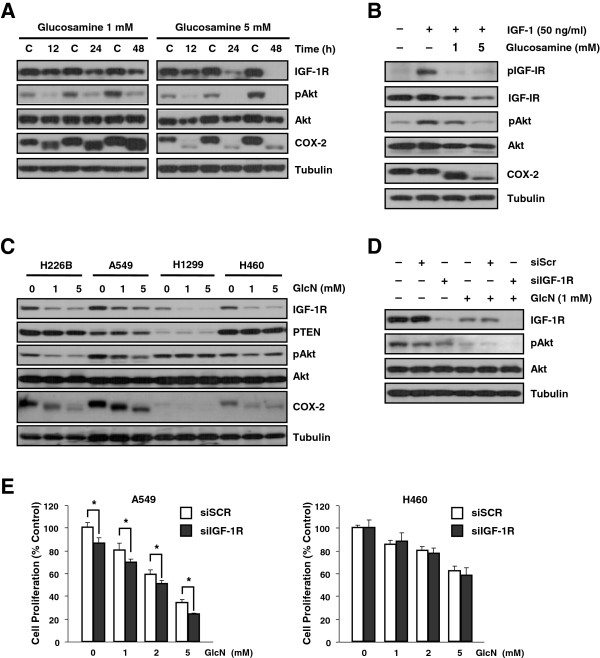
**Glucosamine down-regulates the IGF-1R kinase-dependent Akt pathway in NSCLC cells. (A)** Time- and dose-dependent effects of glucosamine in A549 cells. **(B)** A549 cells were pretreated with glucosamine for 1 day and then activated with IGF-1 for 15 minutes. **(C)** The effect of glucosamine on different NSCLC cell lines. **(D)** A549 cells transfected with siIGF-1R or scrambled non-targeting siRNA (siSCR) 2 days prior to a 1-day treatment with 1 mM glucosamine. **(E)** Cell proliferation assay in NSCLC cells transfected with siIGF-1R and treated with the indicated doses of glucosamine for 2 days. * P < 0.05 compared with siSCR-transfected cells.

Because we observed that glucosamine downregulated CDK4 expression in NSCLC cells (Figure 1D) and a previous report showed that the PI3K/Akt pathway affects CDK4 expression [21], we tested the effect of glucosamine on the IGF-1R/Akt signaling pathway. Glucosamine reduced the IGF-1R and pAkt levels in A549 and H460 cell lines in a dose-dependent manner (Figure 2A). Moreover, activation of both pIGF-1R and pAkt by IGF-1 was downregulated by glucosamine (Figure 2B). These results demonstrate that glucosamine effectively inhibits IGF-1R/Akt signal transduction.

All cell lines showed a dose-dependent decrease in IGF-1R expression, but there was a significant reduction in pAkt expression in the A549 and H226B cell lines (Figure 2C). To confirm that glucosamine inhibited the IGF-1R/Akt signaling pathway, we also carried out small interfering RNA (siRNA) transfection studies. IGF-1R expression was completely abolished following treatment of siIGF-1R–transfected A549 cells with glucosamine. Similarly, pAkt expression was completely abolished in cells cotreated with glucosamine and siIGF-1R (Figure 2D).

We next performed MTT assay on NSCLC cells to determine whether a combination of siIGF-1R and glucosamine inhibits cell proliferation more efficiently than either agent alone. As expected, IGF-1R knockdown enhanced the glucosamine-induced inhibition of cell growth in the A549 cell line but not the H460 cell line in which siIGF-1R did not affect the pAkt level (Figure 2E). Thus, we concluded that glucosamine inhibits the proliferation of NSCLC cells by reducing the expression of IGF-1R, and the extent of the glucosamine-induced reduction in the pAkt level is associated with the anticancer effect of glucosamine.

### Glucosamine and other IGF-1R–targeting agents have similar effects in glucosamine-sensitive and -resistant cell lines

**Figure 3:**
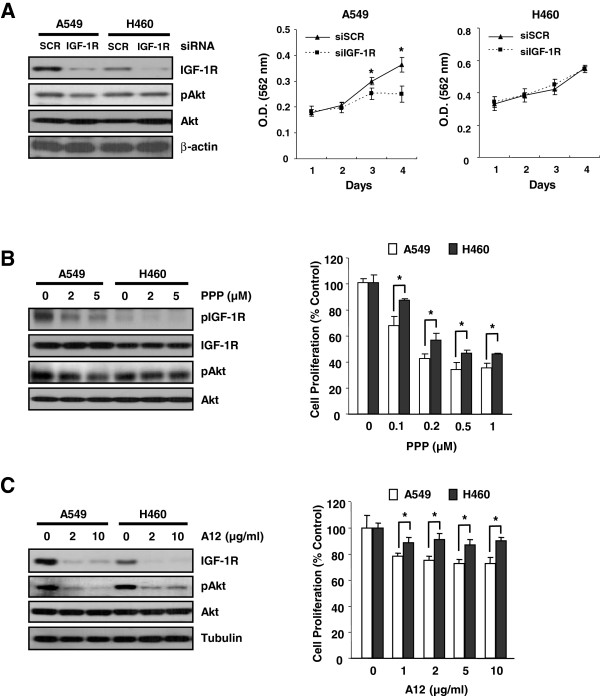
**The inhibitory effect of glucosamine and IGF-1R targeting agents on the IGF-1R/Akt pathway. (A)** The effect of knocking down IGF-1R on the pAkt level and cell growth. **(B and C)** Western blotting (left) and MTT assay (right) of A549 and H460 cells treated with PPP **(B)** and A12 **(C)** at the concentrations indicated. *P < 0.05 denotes significant differences between the conditions indicated.

We hypothesized that if glucosamine acts as an IGF-1R-specific inhibitor, siIGF-1R and other agents that inhibit IGF-1R will exhibit anticancer effects similar to those induced by glucosamine in the A549 and H460 cell lines. Thus, we investigated whether molecules inhibiting IGF-1R also reduce the pAkt level and inhibit cell proliferation in these cell lines. First, siIGF-1R dramatically reduced the IGF-1R level in A549 and H460 cells but only partially reduced the pAkt level in the A549 cell line. In addition, an antisense oligonucleotide targeting IGF-1R only inhibited the growth of the A549 cells (Figure 3A). In addition to siIGF-1R, picropodophyllin (PPP), an IGF-1R-specific small-molecule inhibitor, reduced the levels of pIGF-1R and pAkt and inhibited the growth of A549 cells more efficiently than that of H460 cells (Figure 3B).

One of IGF-1R blocking antibodies, A12, binds directly to IGF-1R and promotes its internalization and degradation [22]. A12 significantly reduced the level of IGF-1R in both A549 and H460 cells (Figure 3C). The pAkt levels were dramatically reduced in the A549 cell line but only slightly reduced in the H460 cell line. In concordance with these results, A12 reduced the proliferation of A549 cells but had no effect on the growth of H460 cells (Figure 3C). These results suggest that IGF-1R is one of the major protein targets of glucosamine in various types of cancer cells that have an IGF-1R-dependent Akt signal transduction pathway.

### Constitutive activation of Akt1 alleviates the growth-inhibitory effect of glucosamine in H226B human NSCLC cells

**Figure 4:**
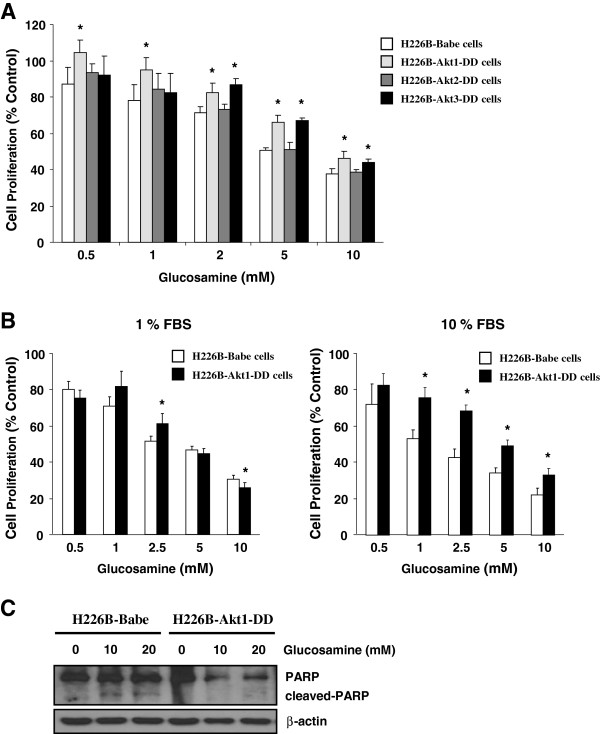
**The role of Akt1 in the glucosamine-induced regulation of cell proliferation in H226B cells. (A)** The indicated cells were incubated with 0.5 ~ 10 mM glucosamine for 3 days and the MTT assay was performed. **(B)** An MTT assay was carried out under low and high serum conditions. *P < 0.05 compared with H226B-Babe cells. **(C)** Adherent and floating cells were analyzed using western blotting.

To evaluate whether constitutive activation of Akt isoforms alters the anti-proliferative effect of glucosamine, H226B-Babe and H226B-Akt1-DD cells were treated with various concentrations of glucosamine for 3 days. Glucosamine effectively suppressed the proliferation of H226B-Babe cells and, to a lesser extent, the proliferation of H226B-Akt1-DD cells (Figure 4A). Under normal culture conditions (10% FBS), 10 mM glucosamine reduced the viability of H226B-Babe and H226B-Akt1-DD cells to 22.1% ± 3.6% and 32.9% ± 3.7% viable cells, respectively. Interestingly, the changes in cell viability were more pronounced in the cells treated under normal culture conditions than in those grown in media containing 1% FBS (Figure 4B). PARP cleavage also was not detected in H226B-Akt1-DD cells exposed to glucosamine (Figure 4C), and we detected only minimal induction of PARP cleavage in H226B-Babe cells treated with 10 and 20 mM glucosamine. These results suggest that constitutive activation of Akt1 may inhibit the anti-proliferative effect of glucosamine.

### Glucosamine affects the stability of IGF-1R in a post-translational modification and proteasome-dependent manner

**Figure 5:**
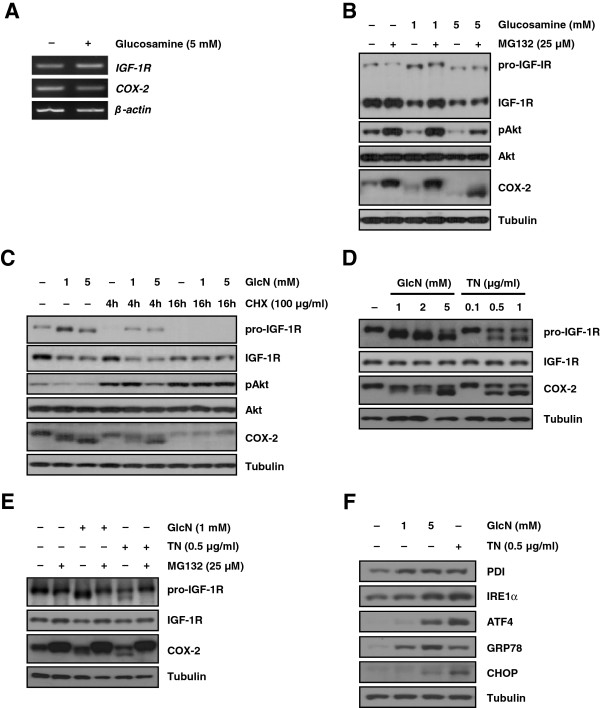
**Glucosamine inhibits IGF-1R at the post-translational level. (A)** Semiquantitative RT-PCR analysis of IGF-1R and COX-2 mRNA levels in A549 cells. **(B)** A549 cells were treated with the indicated doses of glucosamine and the proteasome inhibitor MG132 for 1 day. **(C)** A549 cells were pretreated with the indicated concentrations of glucosamine for 16 hours and then with cycloheximide for 2 hours. **(D)** A549 cells were treated with the indicated concentrations of glucosamine or tunicamycin for 6 hours. **(E)** A549 cells were treated with 1 mM glucosamine or 0.5 μg/ml tunicamycin for 12 hours in the absence or presence of 25 μM MG132. **(F)** Western blot analysis of the expression of various ER stress- and unfolded protein response-related genes was performed using cell lysates from harvested A549 cells.

We next investigated whether the suppression of IGF-1R/Akt signal transduction by glucosamine occurred at the transcriptional and/or translational level. First, we observed that glucosamine treatment did not change the levels of either IGF-1R or COX-2 mRNA (Figure 5A). These findings led us to examine whether the observed decrease in the IGF-1R protein level following exposure to glucosamine was associated with the stability of the IGF-1R protein. The proteasome inhibitor MG132 restored the IGF-1R level in cells treated with 1 mM glucosamine but not in cells treated with 5 mM glucosamine. In contrast, pAkt expression was fully rescued in cells treated with 1 mM glucosamine (Figure 5B). A previous study reported that glucosamine accelerated the proteasome-dependent degradation of only the higher molecular weight species of COX-2; [13] however, our results showed that both higher and lower molecular weight species of COX-2 were restored when cells were treated with either 1 mM or 5 mM glucosamine. In addition to COX-2, the molecular mass of prototype IGF-1R (pro-IGF-1R) was also reduced by glucosamine in a dose-dependent manner (Figure 5B). We next explored whether the glucosamine-induced decrease in the level of IGF-1R protein involved the translation process. Cycloheximide, a ribosomal inhibitor, inhibited the *de novo* biosynthesis of pro-IGF-1R in A549 cells, and glucosamine did not affect the IGF-1R, pAkt, and COX-2 levels (Figure 5C). These findings collectively suggest that glucosamine may induce the hypoglycosylation of pro-IGF-1R and COX-2 and facilitate their degradation at the post-translational level.

Besides, more recent studies have shown that glucosamine inhibits *N*-glycosylation of certain proteins including COX-2, glucose transporter1, and a lipoprotein apo-B-100 [13,23,24]. Therefore, we next tested whether glucosamine induces abnormal *N*-glycosylation of pro-IGF-1R protein. As shown in Figure 5D, glucosamine treatment obviously prevented pro-IGF-1R glycosylation in concentration dependent manner, resulting in low molecular mass of that. Tunicamycin (TN), the protein *N*-glycosylation inhibitor, was used as a positive control to confirm the effect of glucosamine on pro-IGF-1R *N*-glycosylation. We next challenged whether glucosamine-induced abnormal glycosylation of pro-IGF-1R protein is recovered by MG132. As depicted in Figure 5E, reduction of pro-IGF-1R molecular mass by glucosamine was remarkably restored by treating A549 cells with MG132. In addition, previous studies have elucidated that glucosamine caused endoplasmic reticulum (ER) stress and activated a series of signaling pathway termed the unfolded protein response (UPR) [3,25]. We also confirmed that glucosamine induces ER stress and activates the UPR through changing of various marker genes including spliced XBP1, PDI, IRE1α ATF4, GRP78, and CHOP (Figure 5F and Additional file 3: Figure S3). Overall, these data demonstrate that glucosamine negatively affects IGF-1R and COX-2 protein stability through a proteasome-dependent pathway, and the production of hypoglycosylated pro-IGF-1R by glucosamine is associated with this pathway.

### Glucosamine suppress primary tumor growth *in vivo*

**Figure 6:**
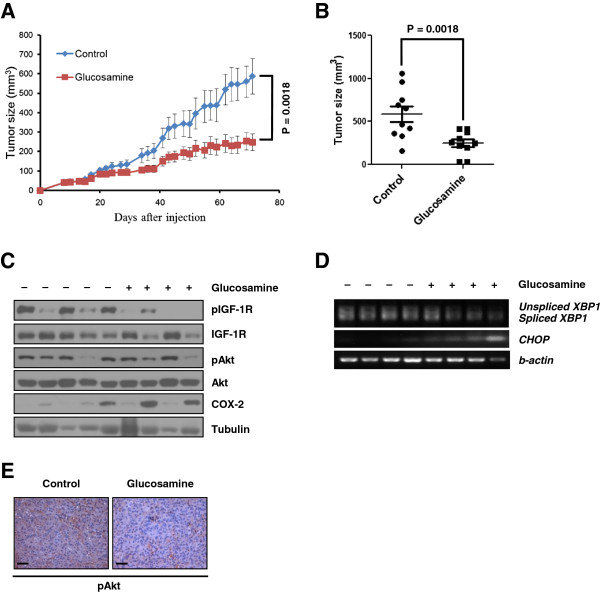
**Glucosamine represses primary tumor growth *in vivo*. (A)** Xenograft experiment for anticancer activity of glucosamine was performed. A549 cells were injected subcutaneously into the BALB/c nude mice. **(B)** Distribution histogram represents the individual signals of mice in each group at day 71. **(C - D)** Tumor tissues extracted from each mice were used for western blotting **(C)** and RT-PCR **(D). (E)** Immunohistochemistry of phosphorylated Akt (pAkt). Bars, 50 μm.

To determine whether glucosamine inhibits primary tumor initiation and growth *in vivo*, glucosamine-sensitive A549 cells were injected subcutaneously into immunocompromised mice. After injection, tumor bearing mice were treated with PBS or glucosamine intraperitoneally. As shown in Figure 6A, glucosamine significantly decreased subcutaneous tumor growth. Although glucosamine did not totally suppress the tumor growth, outgrowth of tumor mass was effectively reduced by glucosamine treatment (Figure 6B). pIGF-1R level significantly was decreased in glucosamine treated tumor tissues compared with PBS treated samples (Figure 6C). We found that glucosamine treated primary tumor showed moderately reduced pAkt level (Figure 6E), although the difference in western blot analysis was not significant (Figure 6C). In addition, RT-PCR data showed that glucosamine induced ER-stress in tumor tissue (Figure 6D). These findings suggest that glucosamine can inhibit primary tumor formation *in vivo* as well as cell proliferation *in vitro* through restraining the IGF-1R/Akt signaling by glucosamine-induced ER-stress.

## Discussion

In this study, we showed that glucosamine effectively inhibits IGF-1R-mediated Akt signal transduction in various human carcinoma cell lines by both suppressing IGF-1-induced IGF-1R activation and reducing IGF-1R protein stability.

Some investigators have reported that glucosamine induces cell-cycle arrest at the G0/G1 phase in human cancer cells [26,27]. These studies have shown that this phenomenon is mediated by decreased cyclin D1 expression and increased p21^waf1/cip1^ expression. Here, we showed that in three NSCLC cell lines, glucosamine could also downregulate CDK4 and CDK2 expression and that the extent of the glucosamine-mediated inhibition of these proteins reflected the proportion of cells arrested in the G0/G1 phase (Figure 1B and D). In addition, Lee et al. reported that expression of CDK4 is associated with PI3K/Akt and that the PI3K inhibitor LY294002 decreases the CDK4 level in corneal endothelial cells [21]. In our study, the pAkt level was more effectively reduced by glucosamine in A549 and H226B cells, which exhibited more significant decreases in CDK levels than either the H1299 or H460 cells (Figure 2C and Figure 1D). Similarly, because pAkt is a positive regulator of cell survival and anti-apoptotic events, the glucosamine-induced increase in apoptosis and cleaved PARP were more evident in A549 cells than in the other cell lines (Figure 1C and D).

Interestingly, we found no significant differences in the total pAkt level in primary tumors derived from glucosamine treated animal although pIGF-1R level was significantly decreased (Figure 6C). However, we also confirmed the decrease of Akt activation in glucosamine treated tissue using histology analysis (Figure 6E). These conflicting findings were probably resulted from drug delivery system. Until 40 days after injection, glucosamine definitely reduced the primary tumor growth. However, after that, the effect of glucosamine was decreased (Figure 6A) and COX-2 level rather increased in the tumor mass (Figure 6C). According to tumor mass getting bigger and bigger, it is difficult that glucosamine could not penetrate into primary tumor. Thus, insufficient dose of glucosamine may partially decrease pAkt level. In addition, the complex tumor microenvironment and the presence of multiple redundant survival pathways at the primary tumor site may reduce the effect of glucosamine and compensate the pAkt activation.

Exogenous glucosamine can be transported across the hydrophobic cell membrane through facilitative glucose transporters (GLUTs) [8,28]. Once inside the cell, glucosamine is converted to UDP-N-acetylglucosamine (UDP-GlcNAc), the substrate for O-GlcNAc modification, through the hexosamine pathway [8]. UDP-GlcNAc covalently modifies cytosolic and nuclear proteins, influencing the stability, localization, enzymatic activity, protein-protein interactions, and phosphorylation status of target proteins [29]. Thus, intact O-GlcNAc modification is very important for proper cell cycle progression, and increasing O-GlcNAc modification of cell cycle regulators using the O-GlcNAcase inhibitor PUGNAc causes growth inhibition that is similar to G2/M arrest [30]. Alteration of glycosylation on the surface of target proteins following glucosamine treatment could be responsible for the decreased CDK2 and CDK4 expression and increased cell cycle arrest. O-GlcNAc modification can also affect the protein turnover rate, and modified proteins are subject to proteasome degradation [31]. We demonstrated that glucosamine influenced the IGF-1R protein stability and facilitated its proteasomal degradation (Figure 5B and C); therefore, the reduction in the IGF-1R half-life following glucosamine treatment may result from the alteration of glycoconjugate structures through O-linked glycosylation. Recent studies have shown that glucosamine inhibits COX-2 *N*-glycosylation and increases the COX-2 turnover rate [13]. In this study, similar effects were also observed for the IGF-1R prototype, which showed a reduced molecular mass (Figure 5).

Although our results suggest that glucosamine effectively inhibits cell proliferation and tumor growth in A549, some of NSCLCs such as H460 relatively show glucosamine-resistant phenotype. Because the IGF-1R/Akt signaling axis includes many signal regulators, such as PI3K, PTEN, and p53, that can influence the pAkt level, [32-35] reduction of pAkt by glucosamine could affect each cell line differently. Therefore, we will investigate whether mutation status of these genes affect glucosamine sensitivity in various types of cancer cell including NSCLCs.

## Conclusions

In summary, our results indicate that glucosamine is an effective inhibitor of the IGF-1R/Akt pathway. The findings of the present study provide evidence supporting the value of glucosamine as an effective and non-toxic IGF-1R blocking agent for cancer therapeutics.

## Competing interests

The authors declare that they have no competing interests.

## Authors’ contributions

All authors participated in design of the study. K-HS, J-HK, and H-YM performed the experimental work and wrote the manuscript. J-KW, J-SN, H-YL, and S-YK contributed to data analysis and interpretation. S-HO conceived of the study, participated in the experimental design, and helped to draft the manuscript. All authors read and approved the final manuscript.

## Pre-publication history

The pre-publication history for this paper can be accessed here:

http://www.biomedcentral.com/1471-2407/14/31/prepub

## Supplementary Material

Additional file 1: Figure S1 Differential effect of glucosamine-induced apoptosis in A549 and H1299 cells. A549 and H1299 cells were treated with 1mM glucosamine for 48 hrs. Cells were stained with Annexin V-FITC and PI and then analyzed by flow cytometry. Results shown are representative of three independent experiments. Click here for file

Additional file 2: Figure S2 Relationship between glucosamine-induced growth inhibition and TGase 2 expression in NSCLC cells. NSCLC cells were grown in RPMI medium 1640 containing 10% FBS for 2 days. Western blot analysis of the expression of TGase 2 and COX-2 was performed using cell lysates from harvested cells. Click here for file

Additional file 3: Figure S3 Glucosamine induce ER stress- and unfolded protein response. A549 cells were treated with glucosamine and tunicamycin for 6 hours. RT-PCR analysis was conducted with indicated gene specific primers. Click here for file
