# Supplementary figures and images for: The novel IGF-IR/Akt–dependent anticancer activities of glucosamine
Source: BMC Cancer. 2014 Jan 20;14:31. doi: 10.1186/1471-2407-14-31 (PMC3901559; doi:10.1186/1471-2407-14-31)

## Slide 1
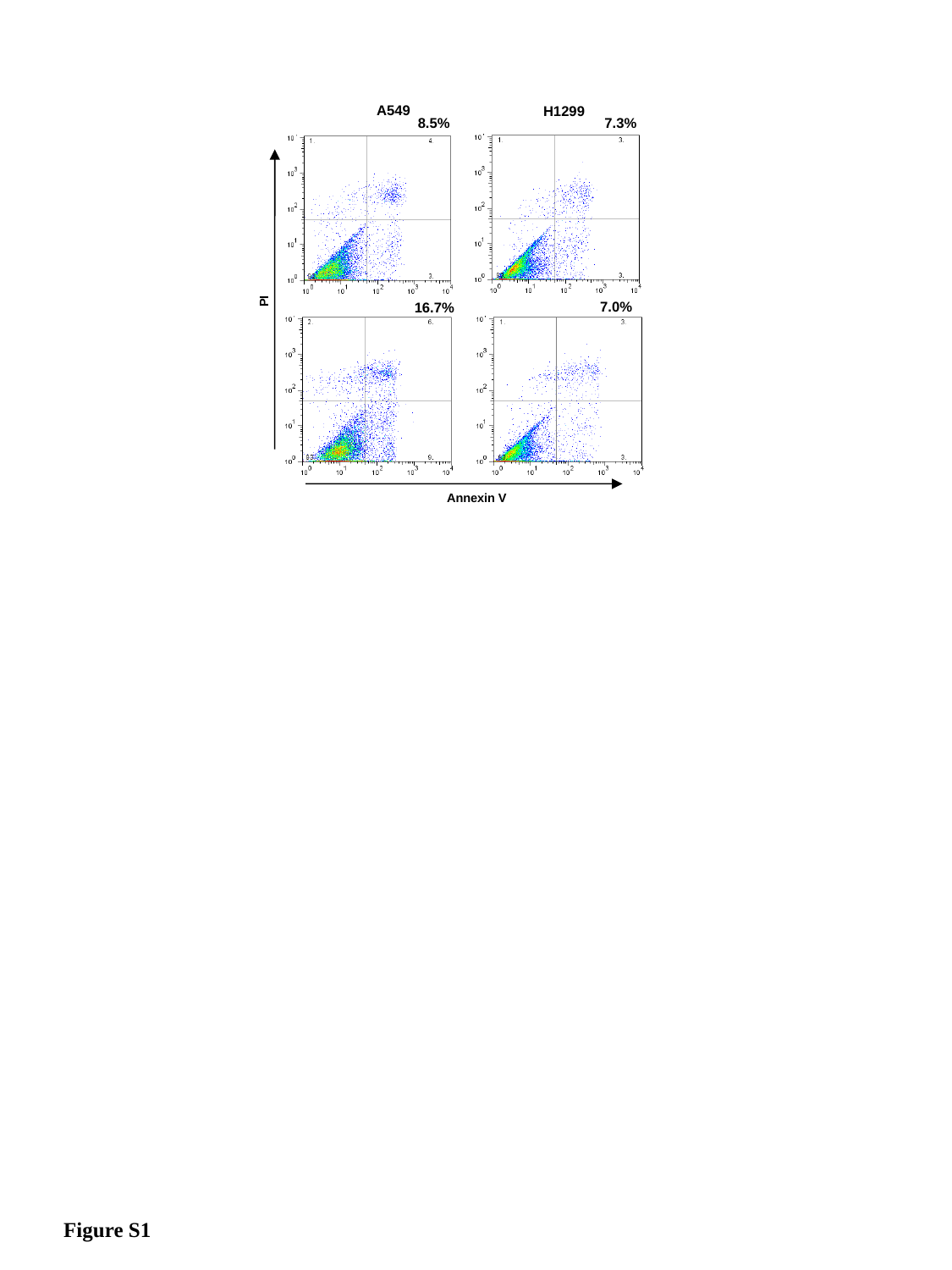

A549
H1299
8.5%
7.3%
7.0%
16.7%
PI
Annexin V
Figure S1

Supplement: Additional file 1: Figure S1 — Differential effect of glucosamine-induced apoptosis in A549 and H1299 cells. A549 and H1299 cells were treated with 1mM glucosamine for 48 hrs. Cells were stained with Annexin V-FITC and PI and then analyzed by flow cytometry. Results shown are representative of three independent experiments. [file 1471-2407-14-31-S1.ppt]

## Slide 1
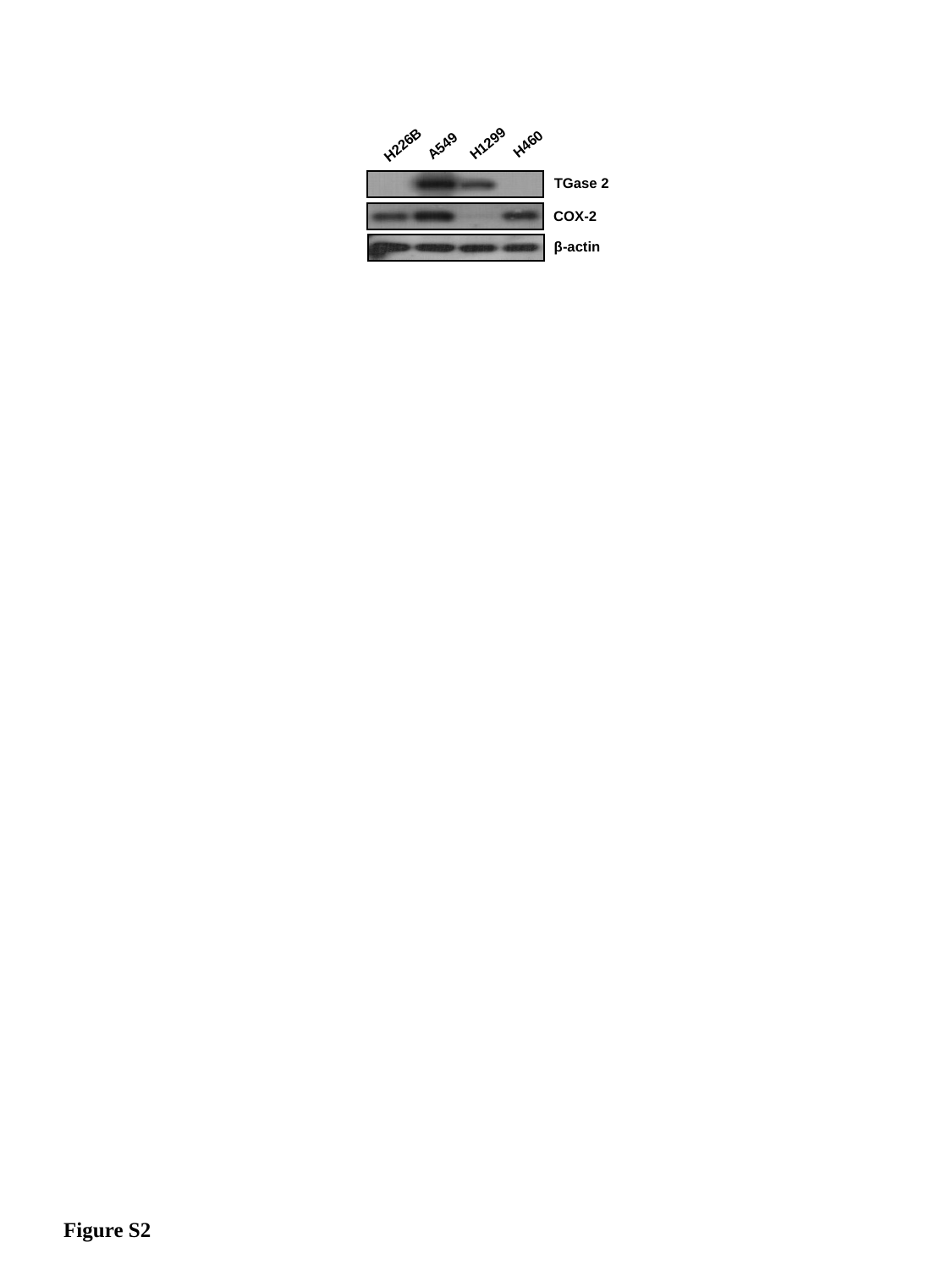

H1299
H460
H226B
A549
TGase 2
COX-2
β-actin
Figure S2

Supplement: Additional file 2: Figure S2 — Relationship between glucosamine-induced growth inhibition and TGase 2 expression in NSCLC cells. NSCLC cells were grown in RPMI medium 1640 containing 10% FBS for 2 days. Western blot analysis of the expression of TGase 2 and COX-2 was performed using cell lysates from harvested cells. [file 1471-2407-14-31-S2.ppt]

## Slide 1
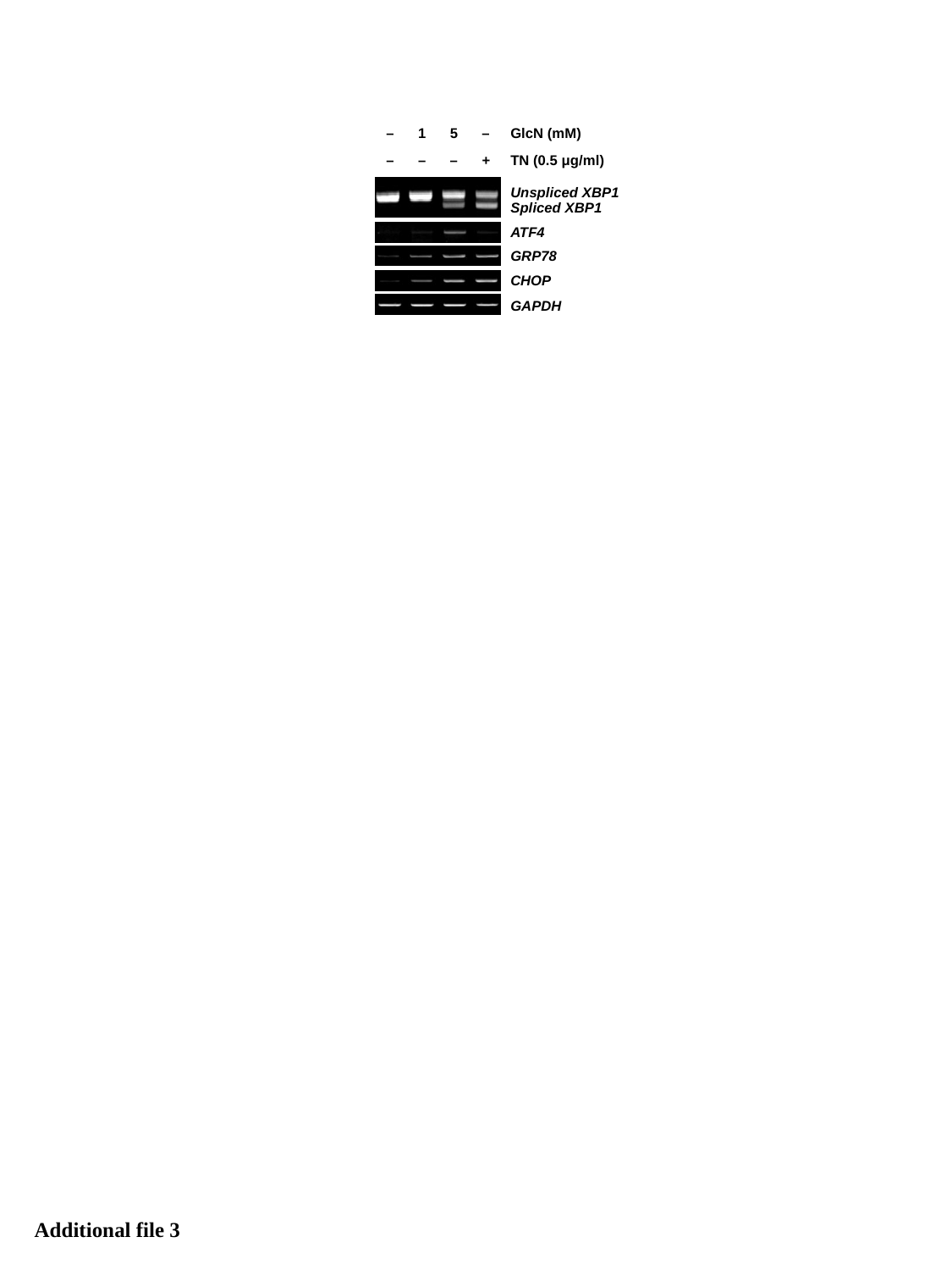

– 1 5 –
GlcN (mM)
– – – +
TN (0.5 μg/ml)
Unspliced XBP1
Spliced XBP1
ATF4
GRP78
CHOP
GAPDH
Additional file 3

Supplement: Additional file 3: Figure S3 — Glucosamine induce ER stress- and unfolded protein response. A549 cells were treated with glucosamine and tunicamycin for 6 hours. RT-PCR analysis was conducted with indicated gene specific primers. [file 1471-2407-14-31-S3.ppt]
